# Which of the acupuncture treatment regimen for lumbar disc herniation is more effective and safer: A protocol for systematic review and network meta-analysis

**DOI:** 10.1097/MD.0000000000025199

**Published:** 2021-03-26

**Authors:** Xiaoying Zhong, Jiaxin Liu, Yanping Wang, Linzi Zhang, Honglai Zhang

**Affiliations:** a School of Medical Information Engineering; b Medical College of Acu-Moxi and Rehabilitation, Guangzhou University of Chinese Medicine, Guangzhou; c Evidence-based Medicine Research Centre, Jiangxi University of Traditional Chinese Medicine, Jiangxi, China.

**Keywords:** acupuncture, lumbar disc herniation, network meta-analysis, protocol

## Abstract

**Introduction:**

Lumbar disc herniation (LDH) is the most common cause of low back pain and severely affects people's quality of life and ability to work. Although many clinical trials and medical reports conducted over the years have shown that acupuncture treatments are effective for LDH, the comparative effectiveness of these different acupuncture therapies is still unclear. This protocol of a network meta-analysis was designed to compare the effects and safety of acupuncture treatment regimens on LDH using both direct and indirect evidence.

**Methods and analysis:**

This protocol is reported according to the 2015 PRISMA-P and PRISMA guidelines for acupuncture. Eight databases and two platforms will be searched for articles published from their establishment to 1 December 2020 with medical subject heading terms and keywords. Three reviewers will verify the eligible randomized controlled trials independently. NoteExpress (3.2.0) software will be utilized to manage the literature. The overall quality of evidence will be evaluated by Confidence In Network Meta-Analysis (CINeMA). Additionally, we will conduct a meta-analysis of the effectiveness, recurrence rate, and symptom score of acupuncture in treating LDH using Review Manager (RevManV.5.4.1) and R4.0.2 software (The R Foundation for Statistical Computing).

**Results:**

The results of the study will be published in journals or relevant conferences.

**Conclusion:**

This proposed systematic review will evaluate the comparative efficacy and safety of various acupuncture methods and combination protocols for LDH.

**Strengths and limitations of this study**

1. This research is the first network meta-analysis and systematic review aiming to investigate the effectiveness and safety of different acupuncture therapies in patients with lumbar disc herniation.
2. Due to the clinical specificity of acupuncture, a descriptive analysis will be conducted of the factors that may produce heterogeneous treatment outcomes in the included studies where possible.
3. In this study, the overall quality of evidence will be evaluated through CINeMA, which will help us better determine the best clinical plan.
4. Problems such as the low-quality clinical randomized controlled trials and the insufficient data being insufficient may prevent us from revealing a high level of evidence-based evidence.

## 1 Introduction

Low back pain has become a widespread global health problem; a study of the global disease burden of low back pain conducted in 2019 showed that there are 568 million people worldwide with low back pain.^[1]^ Approximately two-thirds of the countries assessed in the study had a high incidence of low back pain. The most common cause of low back pain is intervertebral degeneration, which leads to degenerative disc disease and lumbar disc herniation (LDH).^[2]^ LDH is thus the most common cause of low back pain and severely affects the quality of life of patients and their ability to work. In severe cases, LDH may lead to difficulty walking or even paralysis.^[3]^ Thus, it is very important to determine how to appropriately treat LDH. The common symptoms of LDH are lumbago and leg pain, numbness of the lower extremities, and radiating pain. Clinically, most patients choose conservative treatment for LDH, and only 10% to 20% of patients need surgery.

The vast majority of patients with LDH exhibit improved clinical symptoms and functions after systematic treatment, but there is also the possibility of recurrence.^[4,5]^ In recent years, acupuncture has been widely accepted as an auxiliary therapy worldwide.^[6]^ Relevant studies have shown that acupuncture can relieve the pressure in nerves and affect the structural relationship between nerves and lumbar intervertebral discs by stimulating the nerve trunk to alleviate the symptoms of sciatica. Acupuncture also activates nerve fibers associated with the release of endorphins and increases serotonin levels in the brain.^[7–9]^ However, at present, the best acupuncture treatment for LDH has not be identified in China or other countries, and there is a lack of high-quality, sufficient, detailed, and strong evidence. There are also many gaps in the relevant comprehensive studies that need to be explored further.^[10–12]^ By integrating direct and indirect evidence from a network of randomized controlled trials (RCTs), a network meta-analysis (NMA) compares multiple treatments in a single analysis, quantifies different interventions for the same disease, and prioritizes the relative merits of outcome indicators to determine the optimal regimen. This method is appropriate for assessing the unique clinical pathway characteristics of traditional Chinese medicine.^[13,14]^

Therefore, we aim to identify the safest and most effective treatment options for LDH through a systematic review and NMA of the literature on different forms of acupuncture treatments for LDH.

## 2 Materials and methods

### 2.1 Study registration

The study is based on the PRISMA-P^[15]^ and PRISMA checklists for acupuncture.^[16]^ This protocol was registered on February 24, 2021 (http://inplasy.com/). The registration number is INPLASY202120077.

### 2.2 Inclusion and exclusion criteria for study selection

#### 2.2.1 Inclusion criteria

1. Studies will be included if they enrolled adults (≥18 years old) who were diagnosed with LDH by computed tomography or magnetic resonance imaging.
2. Trials will be included which assessed acupuncture therapies for LDH including electroacupuncture, warm acupuncture, fire acupuncture, manual acupuncture, acupuncture point embedding, acupuncture point application, and heat-sensitive moxibustion. No restrictions regarding the type of needle, moxibustion supplies, or treatment sessions were used.
3. Studies will also be included if the control group received a guideline-recommended treatment, such as drugs, surgery, and kinesitherapy.
4. The study was an RCT published in Chinese or English.
5. The primary outcomes were overall effectiveness, function, and pain relief, and the secondary outcomes were recurrence rate, quality of life, muscle thickness, and the incidence of adverse events.

#### 2.2.2 Exclusion criteria

1. Trials with unclear diagnostic criteria.
2. Trials with unclear or inconsistent end-point indicators.
3. Trials with fewer than 30 experimental cases in a single group will be excluded. Literature reviews and duplicate studies will also be excluded.

### 2.3 Information sources (databases)

#### 2.3.1 Data sources

**Figure 1:**
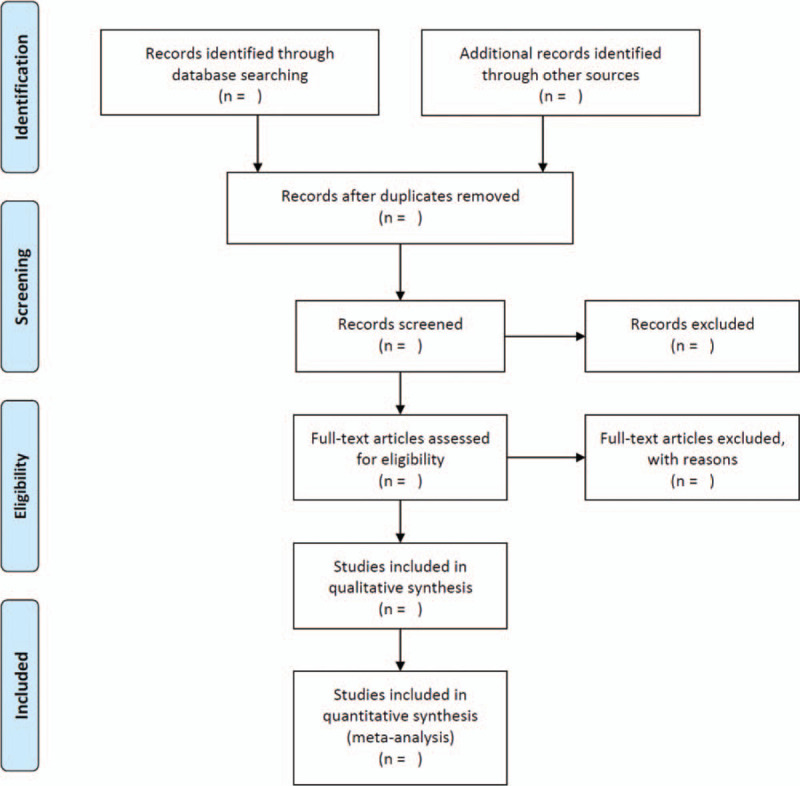
PRISMA flow diagram of the study selection process.

We will systematically search for articles in 8 databases, including PubMed, Embase, the Cochrane Library, Web of Science, the Chinese Biomedical Literature Database (http://www.sinomed.ac.cn/), Chinese National Knowledge Infrastructure (https://www.cnki.net/), Wan Fang (http://www.wanfangdata.com.cn/index.html), and VIP (http://www.cqvip.com/). Two databases of privately and publicly funded clinical studies will be searched for ongoing RCTs, clinicaltrials.gov and the Chinese Clinical Trial Registry. Articles published from the establishment of the databases to December 1, 2020 will be identified. The PRISMA flowchart (Fig. 1) describes the overall process.^[17]^

#### 2.3.2 Search strategy

We will use a combination of subject and nonsubject terms according to the search systems of the different databases. The search strategy for PubMed is detailed in Table 1. This search strategy will be modified as required for the other electronic databases.

**Table 1:** 1Search strategy used for the PubMed database.

Number	Search items
#1	“Intervertebral Disc Displacement” [Mesh]
#2	“Intervertebral Disc Degeneration” [Mesh]
#3	“Sciatica” [Mesh]
#4	“Acupuncture” [Mesh]
#5	“Acupuncture Therapy” [Mesh]
#6	randomized controlled trial [Publication Type]
#7	controlled clinical trial [Publication Type]
#8	randomized [Title/Abstract]
#9	placebo [Title/Abstract]
#10	randomly [Title/Abstract]
#11	trial [Title/Abstract]
#12	groups [Title/Abstract]
#13	#1 or #2 or #3
#14	#4 or #5
#15	#6 or #7 or #8 or #9 or #10 or #11 or #12
#16	13 and 14 and 15

### 2.4 Data extraction and management

#### 2.4.1 Study selection

Two researchers will independently browse the titles and abstracts of the articles. If necessary, the full texts will be downloaded and read for further evaluation. A third researcher will read the full texts and screen the articles with reference to the inclusion criteria. For each excluded study, reasons for exclusion will be given. If there is a disagreement, a fourth investigator will be consulted to resolve any differences. The titles and first authors of all included studies will also be checked. If there are duplicate studies, the investigators will include the article that was published first.

#### 2.4.2 Data extraction

Two reviewers will independently extract parameters from the applicable studies, including identifying information (the first author and year of publication), essential characteristics (country, language, number of study centers, total sample size), participant characteristics (age, sex), diagnostic indicators, relevant quality assessment indicators (random sequence generation method, allocation hiding method, blinding, completeness of the outcome data, the presence of selective publication bias and other biases), interventions (type of acupuncture, frequency/treatment/time), control treatment measures (including name, dose, frequency, and duration), and outcome measures (good data and time points per measurement or follow-up, adverse effects).

#### 2.4.3 Measures of treatment effect

RevMan (V.5.4.1)^[18]^ and R4.0.2 software will be used to compile the data. Risk ratios with 95% confidence intervals and weighted mean differences or standardized mean differences with 95% CIs will be used to present the dichotomous data and continuous data, respectively.

#### 2.4.4 Dealing with missing data

If the data of interest are unclear or not reported in an article, we will contact the corresponding author of the article by phone or email. If contact is not possible, the trial will be excluded.

#### 2.4.5 Assessment of the quality of the included studies

In this study, the quality of evidence for NMA will be graded using CINeMA, an online networked meta-analysis evidence grading application based on the grading of recommendations, assessment, development and evaluations (GRADE) method, which considers NMA as a whole. The quality of evidence for NMA is graded by considering the following 6 domains: intra-study bias (risk of bias), inter-study bias (publication bias or reporting bias), concordance, imprecision, heterogeneity, and inconsistency. Each of these areas can be classified as not serious (no concern, no downgrade), serious (some concern, 1 downgrade) or very serious (major concern, 2 downgrades) according to their severity, and the final grade for the quality of NMA evidence is selected according to the GRADE system: high, medium, low or very low. CINeMA will be conducted calculate the contribution matrix of the NMA with the netmeta package in R4.0.2 software and judge the levels of intra-study bias and indirectness based on the contribution of each study included in the NMA to the NMA results; based on the comprehensiveness of the literature search, the completeness of previous empirical studies and statistical analysis, the inter-study bias will be judged as “suspicious” (SUSPECTED, downgraded) or “undetected” (UNDETECTED, not downgraded). The rules for determining imprecision and heterogeneity are based on whether the confidence interval or prediction interval contains a null line and a prespecified minimum clinically important difference; inconsistency is determined by the local and network-wide inconsistency test results.

### 2.5 Statistical analysis

A descriptive table will be provided summarizing the key characteristics of each eligible study, including the first author of the article, year of publication, intervention, diagnostic criteria, sample size, primary outcome indicators, and study design. A network diagram will be created to show which intervention categories were compared, with larger network nodes indicating a larger number of patients and thicker connecting lines between nodes indicating a larger number of trials. The meta-analysis will be conducted with RevMan 5.4.1 and R4.0.2 software.

#### 2.5.1 Direct pairwise meta-analysis

First, RevMan5.4.1 will be used to pool the data. We will test the heterogeneity of the included experimental studies. The heterogeneity of each pairwise comparison will be tested by the Chi-Squared test (significance level α = 0.1). If *I*^2^ ≤ 50%, there is no heterogeneity, and a fixed-effects model will be used. If *I*^2^ ≥ 50%, significant heterogeneity exists among the studies in the group, and we will use a random-effects model for the meta-analysis. We will explore the reasons for the existence of heterogeneity in terms of the demographic characteristics of the subjects, the degree of variation in the interventions, and other factors. A sensitivity analysis will also be used to identify the sources of heterogeneity.

#### 2.5.2 Network meta-analysis

We will use the netmeta package of R4.0.2 software to conduct the NMA. The netmeta package is currently the only package that has been developed based on “the frequency distribution” and completes the network meta-analysis in 1 step without using a regression model.^[19]^ Therefore, it prevents artificial bias caused by complex prior settings, initial value settings, and the settings of regression model dummy variables and variance-covariance matrices in Bayesian statistics and simplifies the operator's settings for each parameter. In addition, we will generate a mesh relationship graph for a mesh meta-analysis by the network program package.^[20]^ The grid-relations diagram will clearly present which direct comparisons exist between the interventions and which interventions can be compared indirectly.

#### 2.5.3 Publication bias

In this study, Egger regression test will be used to assess publication bias, which will help avoid observation bias, and the results will be presented as a funnel plot.

#### 2.5.4 Other analyses

Due to the clinical specificity of acupuncture, descriptive analyses of the acupuncturist, acupoint selection, acupuncture technique, amount of electrical stimulation, and any other factors that may produce heterogeneity in the treatment outcomes detailed in the included studies will be conducted where possible.

### 2.6 Patient and public involvement statement

For this systematic evaluation study, ethical approval is not required, as no individuals regarding identifiable individuals are included. The results of the study will be published in journals or relevant conferences.

## 3 Discussion

In clinical practice, acupuncture has been widely used in the treatment of LDH. Due to the lack of comparative effectiveness studies, selecting the best acupuncture regimen is challenging for clinicians. In this systematic evaluation, we will evaluate the comparative efficacy and safety of various acupuncture methods and combination protocols for LDH. An NMA will be conducted to summarize direct and indirect evidence and reliably prioritize acupuncture regimens for the treatment of LDH.^[6]^ As few randomized trials have directly compared different acupuncture methods, this study will provide the highest level of evidence to date through an NMA. This systematic evaluation will also be the first to compare the effectiveness and safety of different acupuncture regimens for LDH. However, there are some limitations of this study. First, medical databases in other languages (e.g., Korean and Japanese) will not be searched because of language barriers, so there may be language bias, which may lead to insufficient data. Second, the quality of the original trials will affect the quality of the summary, so we will strictly control the quality of the included studies and the similarity of their essential characteristics. Our review will provide clinicians with reliable evidence on acupuncture treatment protocols for LDH and encourage the widespread clinical use of acupuncture for LDH.

## Author contributions

**Conceptualization:** Honglai Zhang.

**Funding acquisition:** Honglai Zhang.

**Methodology:** Xiaoying Zhong, Jiaxin Liu.

**Project administration:** Xiaoying Zhong.

**Software:** Xiaoying Zhong, Jiaxin Liu, Linzi Zhang.

**Writing – original draft:** Xiaoying Zhong, Jiaxin Liu.

**Writing – review & editing:** Yanping Wang, Honglai Zhang.
